# Gold Nanoparticles in Conjunction with Nucleic Acids as a Modern Molecular System for Cellular Delivery

**DOI:** 10.3390/molecules25010204

**Published:** 2020-01-03

**Authors:** Anna Graczyk, Roza Pawlowska, Dominika Jedrzejczyk, Arkadiusz Chworos

**Affiliations:** Centre of Molecular and Macromolecular Studies, Polish Academy of Sciences, Sienkiewicza 112, 90-363 Lodz, Poland; graczyk@cbmm.lodz.pl (A.G.); rozapech@cbmm.lodz.pl (R.P.); djedrzej@cbmm.lodz.pl (D.J.)

**Keywords:** structural RNA, RNAi, AuNP, gene expression regulation, cellular uptake

## Abstract

Development of nanotechnology has become prominent in many fields, such as medicine, electronics, production of materials, and modern drugs. Nanomaterials and nanoparticles have gained recognition owing to the unique biochemical and physical properties. Considering cellular application, it is speculated that nanoparticles can transfer through cell membranes following different routes exclusively owing to their size (up to 100 nm) and surface functionalities. Nanoparticles have capacity to enter cells by themselves but also to carry other molecules through the lipid bilayer. This quality has been utilized in cellular delivery of substances like small chemical drugs or nucleic acids. Different nanoparticles including lipids, silica, and metal nanoparticles have been exploited in conjugation with nucleic acids. However, the noble metal nanoparticles create an alternative, out of which gold nanoparticles (AuNP) are the most common. The hybrids of DNA or RNA and metal nanoparticles can be employed for functional assemblies for variety of applications in medicine, diagnostics or nano-electronics by means of biomarkers, specific imaging probes, or gene expression regulatory function. In this review, we focus on the conjugates of gold nanoparticles and nucleic acids in the view of their potential application for cellular delivery and biomedicine. This review covers the current advances in the nanotechnology of DNA and RNA-AuNP conjugates and their potential applications. We emphasize the crucial role of metal nanoparticles in the nanotechnology of nucleic acids and explore the role of such conjugates in the biological systems. Finally, mechanisms guiding the process of cellular intake, essential for delivery of modern therapeutics, will be discussed.

## 1. Introduction

Nanomaterials are referred as materials, made of unbound or aggregated particles, of which one or more external dimensions is in the range 1–100 nm (EU statement from 2011—2011/696/EU). Nanoparticles, belonging to the nanomaterials’ group, are defined as objects with three external nanoscale dimensions [1]. Different nanoparticle types have been described thus far, including metal nanoparticles, silica, hydrogels, carbon etc. [1,2]. Depending upon the application, appropriate type, size and shape, nanoparticles may be designed and produced using either chemical or biochemical methods. So-called green nanoparticles represent a trend in nanotechnology, where biological or plant synthesis is used to produce nanoscale elements in a controlled way [3,4].

**Figure 1:**
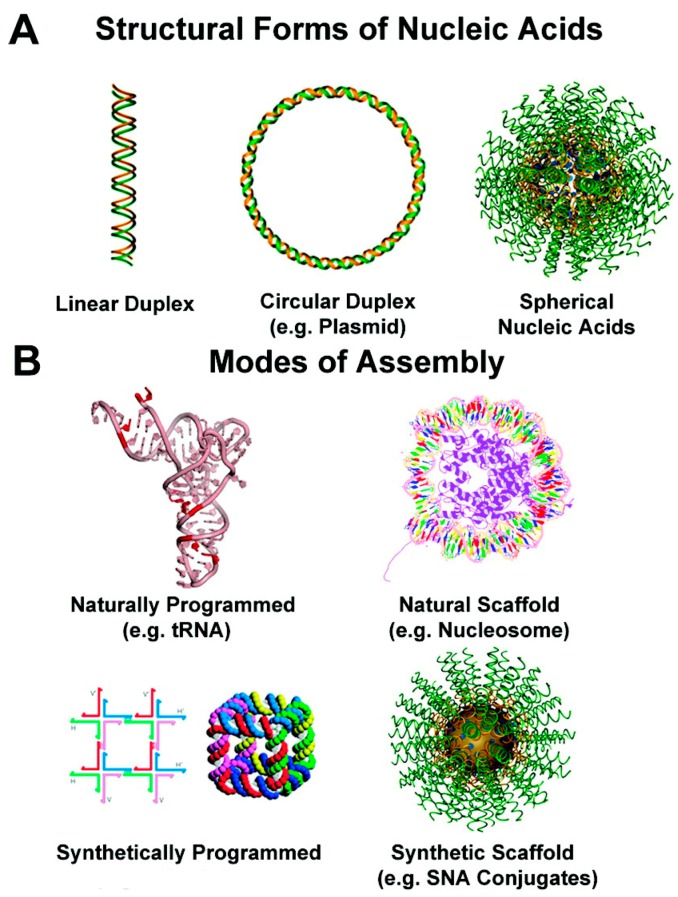
Different forms of nucleic acids. (**A**) Structural forms of nucleic acids: linear or circular duplexes, spherical nucleic acids; (**B**) modes of assembly: native RNA structures, natural scaffolds, synthetically programmed, synthetic scaffolds. Adapted with modification [14].

Another important branch of nanotechnology focuses on binding biomolecules like proteins [5,6] or nucleic acids [7] with nano-objects. In this case nanotechnology helps to manipulate features of the nano-devices by altering components attached to their surface, potentially boosting their performance or bioavailability. Amongst many applications of nanotechnology this approach holds the promise to supplement current methods for imaging, diagnosis and treatment of difficult diseases [8]. Biological elements have been conjugated with the array of nano-objects, including metal nanoparticles, liposomes, hydrogels, graphene, quantum dots, etc. [4]. Metallic nanoparticles have earned their long-lasting recognition due to broad range of their applications in biotechnology or engineering [9]. Current methods allow for synthesis and assembly of modified nanoparticles, with new features or capacity to conjugate biological cargo like drugs, nucleic acids, antibodies etc. (Figure 1). Conjugates of metallic nanoparticles have already been utilized in targeted drug delivery, diagnostics and imaging. For instance, the DNA-gold nanoparticle hybrids, first introduced by Mirkin et al. [10], later applied as a spherical nucleic acids (SNAs) technology has become a brand in clinical biotechnology and a foundation of the Aurasense and later Exicure company (Chicago, IL, USA) [11]. Spherical nucleic acids (SNA) are polyvalent nanostructures, composed of a metal nanoparticle core with nucleic acids fragments attached to the surface [12]. Since then the technology of spherical nucleic acid has been expended into variety of structures [13]. Here, we review the current knowledge regarding arrangements involving metal nanoparticles and nucleic acids, as well as their potential biological applications.

## 2. Metal Nanoparticles in Medicine

**Figure 2:**
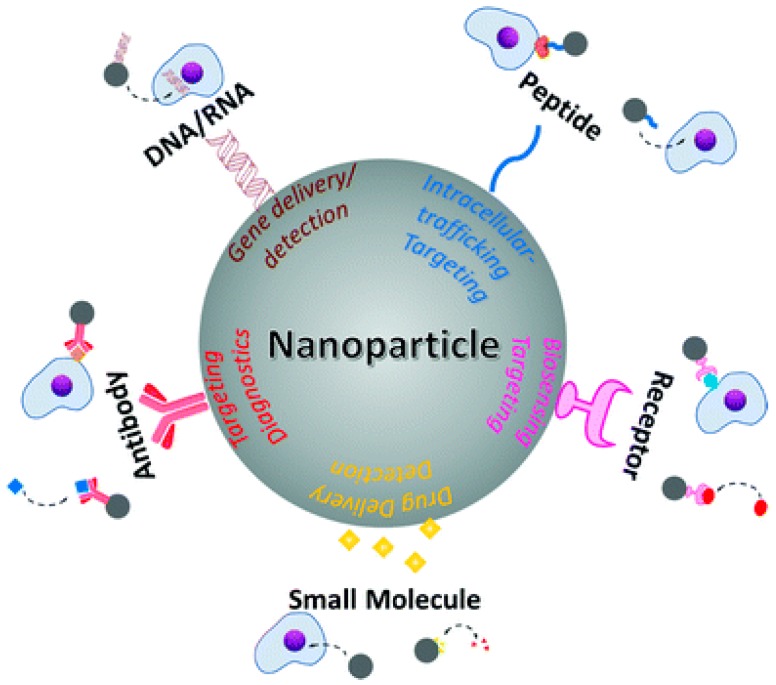
Biomedical applications of nanoparticles through conjugation with various active moieties including nucleic acids, peptides, receptors, antibodies, and small molecules. Taken with permission from [9].

Metallic nanoparticles have been successfully synthetized and utilized in juxtaposition with biomolecules or introduced as components of nanomaterials. Considering therapeutic and diagnostic purposes it is crucial to generate stable, enzyme resistant conjugates with biological functionality. This can be achieved by combining nanoparticles with biologically active elements, like ligands, drugs, antibodies, peptides, nucleic acids etc. (Figure 2). However, to successfully introduce such devices into the biological systems it is essential to use building blocks that are biocompatible and can be synthetized in relatively large quantity (at least gram scale). Metal nanoparticles have been utilized for such approaches, out of which the most commonly studied are the gold, silver, or platinum nanoparticles.

### Gold Nanoparticles

There are several reasons why gold nanoparticles (AuNP) are the most widespread and became a common choice for many studies. The most important are their chemical resistivity, enzymatic stability, low cytotoxicity [15] and physicochemical characteristics (for more information regarding gold nanoparticles and their properties we recommend reviews [16,17]). Currently AuNP can be efficiently synthesized in variety of sizes, stabilized with neutral or charged groups and further functionalized via ligand exchange. Moreover, the plethora of optical traits resulting from surface plasmon of AuNPs and relatively large extinction coefficients made gold an exceptional material for research, engineering, and medical applications [18]. AuNPs are therefore good candidates for in vivo imaging and studies compared to other materials like organic dyes or quantum dots. Taken together, due to the negligible toxicity of gold, and the simplicity of the controlled synthesis of AuNPs and functionalization, it became of interest to utilize AuNPs in conjunction with biomolecules such as nucleic acids [19,20,21], peptides [22], proteins [23,24], antibodies [25,26], and chemotherapeutic agents [27]. An advantage of conjugated AuNPs is improved effectiveness of the active ligands bound to the nanoparticle. Several studies have reported a synergistic effect of the active compound when delivered via AuNP compared to the same compound alone [28,29,30].

Many medical applications speak for the great utility of the gold nanoparticles. For example, owing to their optical properties, AuNPs may be introduced into photothermal therapy [31,32], which may further be developed by coating AuNPs with selective moieties targeting a specific cancerous cell [33,34]. Mentioned above optical properties became of interest in the field of bioimaging, where AuNPs serve as probes to visualize cellular compartments and allow to follow nanoparticles’ cellular uptake, specificity and locations, which might be used for the targeted therapies [26,35,36]. In the greater scale, AuNPs were shown to aid mapping of lymph nodes [37], or vascular blood flow [38]. AuNPs are also used as a tool for cancer imaging and/or a core element for the specific mRNA, creating a dual probe to screen for melanoma, prostate or carcinoma cancer cells, and circulating breast cancer cells [39] and brain tumors [40].

The ease of functionalization allows the delivery of a variety of cargos to the cells. Such a quality is exploited in immunotherapies, where AuNP are conjugated with specific antigens or agents triggering the activation of immune response cells [41,42] or antibodies’ production [43]. Such therapies hold a promise for cancer and infectious diseases treatment. All the methodologies utilizing AuNPs can be used separately but there exist combinatorial approaches that use a mix of techniques in order to boost the therapeutic potential [44]. More detailed description and references to AuNPs applications may be found in the reviews [32,34,39,43,45].

## 3. Templated DNA Structures—DNA Nanotechnology

The deoxyribonucleic acid (DNA) is commonly known for its essential role in storing and transmitting genetic information. However, the DNA nanotechnology gives it much more context and utilizes the structural properties of this natural biopolymer. The scaffolded DNA methodology was initiated by Seeman in late 80s [46]. It led to construction of great number of branched DNA complexes, double- [47,48,49,50], triple- [51,52,53] and paranemic-crossover tiles [54,55], and further developed to two-dimensional (2D) lattices [56], including the “DNA barcode” [57] and pioneering three-dimensional (3D) architectures [58,59]. The outburst of DNA nanotechnology has gained its recognition with Rothemund’s concept of DNA origami [60] that provided the methodology to fold long, single-stranded DNA molecules (originally virus circular genomic DNA from M13mp18) into arbitrary 2D shapes. Further improvements allowed to build complex structures that not only hold desired architecture, but also are able to respond to the environmental changes. DNA nano-machines are capable to rotate by switching between B and Z form at high ionic strength [61], sense the pH [62,63], and respond to introduction of UV light [64]. The DNA “walkers” can be designed on the pre-defined DNA origami pathway [65], based on enzymatic activity [66,67,68] or strand displacement strategy [69,70]. DNA “gears” that “roll” against one another have also been constructed [71]. Moreover, DNA nano-robots [72] and “DNA box” with controllable lid [73] were both reported to deliver and release compounded cargos. Advanced and complex DNA contracts were developed and used for specific intracellular targeting, such as sequence responsive DNA cubes targeting a prostate cancer associated gene [74] and DNazyme motor initiated with specific microRNA [75,76]. The variety of functions may be employed to form highly specific binding sites on DNA scaffold, including specific enzyme-substrate interactions [77], aptamer-protein [78], antibody-protein or protein-protein affinity [79]. For instance biotinylated oligonucleotides were successfully applied to position the streptavidin modified locations on the lattice, leading to DNA-streptavidin conjugates [80] that have found numerous applications [57,81,82,83,84]. Another strategy, based on the specific protein–DNA interaction engaged the Holiday junction binding protein (RuvA) on the DNA lattice [85]. The aptamers had been shown to specifically interact with wide range peptides, proteins, and small molecules [86]. The thrombin-binding aptamer-bearing DNA nanostructures were reported showing the directed assembly of thrombin protein arrays [87,88]. For more insight into the DNA-protein conjugates we recommend reviews [89,90,91,92].

DNA structuralized conjugates can be potentially applied as drug delivery agents, molecular probes for diagnostics, in synthetic biology, studies of interactions and structure of biomolecules, and many others [93,94]. Moreover, such conjugates may also be utilized to create DNA nanomachines with opto-electronic properties for advanced materials [95,96,97,98].

Apart from being used to organize biomolecules, DNA nanostructures can also dictate the assembly of smaller inorganic nanomaterials into two- or three-dimensional DNA structures [99]. Contrary to biomolecular conjugates, chemical deposition is based on the non-specific interactions such as intercalation, DNA major groove binding, electrostatic interactions and metal chemical reduction, that can drive DNA-templated deposition of metallic particles. The semiconductor [100], palladium [101], platinum [102], gold [103], and silver [104] nanoparticles (NPs) and nanowires templated on DNA scaffold have been constructed utilizing this strategy.

### DNA—Metal Nanoparticles Conjugates

**Figure 3:**
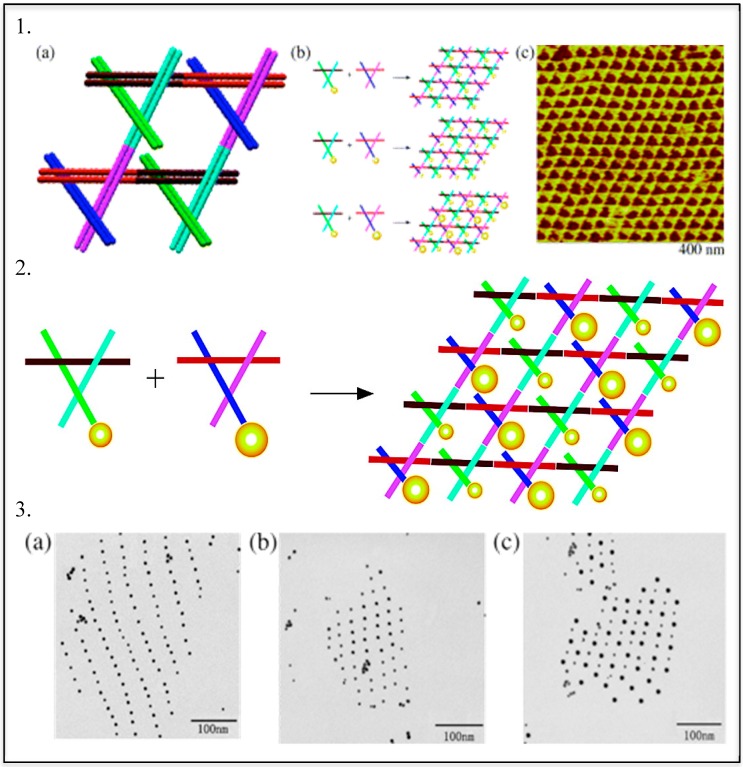
Assembly of two-dimensional (2D) arrays. Taken with permission from [110]. (**1**) (**a**) Where two domains (cyan bonding to magenta and brown bonding to red) are involved in array formation, while the end of the third domain (blue or green) is free to be involved in scaffolding operations; (**b**) nanoparticle attachment (5 nm particles attached to only one of the two triangular tiles, 5 nm particles attached to both of the tiles, and 5 nm particles attached to one of the tiles and 10 nm particles attached to the other tile); (**c**) tapping-mode atomic force micrograph of an underivatized array. (**2**) Schematic formation of 2D DNA-AuNP arrays. (**3**) Transmission electron micrographs of 2D arrays of organized gold nanoparticles. (**a**) An array where one tile contains 5 nm particles. (**b**) An array where both tiles contain 5 nm particles. (**c**) An array where one tile contains a 5 nm particle and the other tile contains a 10 nm particle.

The first examples of DNA-directed assembly of inorganic NPs were described in 1996, when two groups built their constructs utilizing the complementarity of the DNA linker molecules. Alivisatos’ group developed a method to align monovalent nanoparticle conjugates on DNA templates for creation of small, periodic nanoparticle assemblies [105]. Concurrently Mirkin reported the assembly of networked particle arrays composed of polyvalent oligonucleotide-gold nanoparticle conjugates and complementary linkers [10]. Similar strategies have been applied to achieve DNA-directed formation of two- and three-dimensional AuNP assemblies. The AuNPs were successfully patterned on the double-crossover tile arrays via hybridization of oligonucleotide-functionalized nanoparticles and complementary tiles [106,107,108,109]. Later, this approach was employed to arrange the two sizes AuNPs on a triangular shaped DNA scaffold (Figure 3) [110].

In 2008, Mirkin’s and Gang’s groups independently reported DNA-mediated assembly of polyvalent oligonucleotide gold nanoparticle crystals [111,112]. By varying properties of DNA linkers (length, flexibility, etc.), it was possible to put gold nanoparticles location into either body-centered or face-centered cubic unit cells. The number of AuNP crystalline structures grown benefiting from the numerous oligonucleotides modifications. More recently DNA-AuNP formation of crystalline structure was reported [113]. The specificity of sulphur–gold interaction was also widely exploited to mediate the DNA–AuNPs assembly. DNA pyramidal nanocage with four hexanethiol linker-modified vertices directed the assembly of four different AuNPs varying in diameter [114]. DNA tetrahedron with three thiol groups and one biotin group at the vertices was made to assemble both gold substrates and streptavidin molecules [115]. The mixed approach, combining self-assembly, molecular recognition, and templating, relying on an oligonucleotide covalently bound to a high-affinity gold-binding peptide was proposed by LaBean [99]. It allowed assembly in which the peptide location within the self-assembling DNA structure defined the final spatial arrangement.

**Figure 4:**
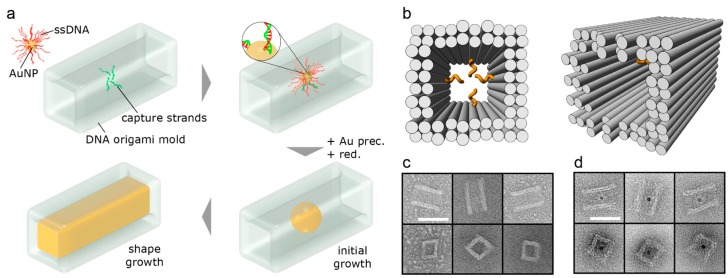
Casting metal nanoparticles (NPs) with prescribed shapes using DNA nanostructure molds. (**a**) Scheme of the nanostructure synthesis. Molds with an inner cavity are fabricated using the DNA origami method. Capture DNA strands in the center of the cavity allow the site-specific introduction of single gold nanoparticle (AuNP) seeds carrying DNA strands with the complementary sequence. In the presence of a gold precursor and a reducing agent, the growth of the nanoparticle is initiated. Further gold deposition is blocked by the mold walls, such that the particle adopts the shape that is dictated by the mold; (**b**) Cartoon of the DNA mold with DNA double-helices depicted as gray cylinders and capture strands as orange spirals; (**c**) TEM images of the mold; (**d**) TEM images of the mold with bound gold nanoparticle seed. In panels c and d, the top and bottom rows show, respectively, views onto a mold side wall and along the cavity axis. All TEM images are shown at equal magnification with the scale bar corresponding to 40 nm. Taken with permission from [119].

The DNA origami structures have also been used for gold nanoparticles organization, for example rectangle shaped DNA origami was engaged to display AuNPs functionalized with lipoic acid-modified DNA [116]. Triangular origami was used to pattern AuNPs of various diameter (5, 10 and 15 nm) in a linear fashion [117]. Similar triangular scaffold was used for site-specific assembly of silver nanoparticles (AgNP) and dimeric AgNP–AuNP nanoarchitectures [118]. In 2014, two groups independently published their strategies to obtain inorganic nanostructures with arbitrarily predefined three-dimensional shapes. The three-dimensional DNA origami nanostructures harboring an internal cavity were used as molds for a nucleating gold “seeds” [119,120]. Employing this method Bathe and Yin’s group showed synthesis of three distinct silver cuboids at three independently tunable dimensions, silver and gold nanoparticles with diverse cross sections, and composite structures with homo- and heterogeneous components at 3 nanometer resolution (Figure 4). Seidel’s group demonstrated the fabrication of 40 nm long rod-like gold particles with quadratic cross section and the formation of higher order assemblies [119].

The advances in DNA nanotechnology led to other applications in biophysics, nanooptics, site-specific chemistry, and plasmonics [94]. DNA nanostructures have already been applied in nanomechanical devices [61,67,69,121], computing systems [122,123,124,125] and programmable/autonomous molecular machines [126,127,128]. Deng and Mao showed that DNA lattice could serve as a mask for lithographic deposition of gold print onto mica, for use in electronics [129]. The molecular printing strategy was used to chemically transfer a discrete pattern of DNA strands from a three-dimensional DNA structure to a gold nanoparticle [130]. It was reported that gold nanoparticles mimicked the DNA sequence configuration encoded in the parent template with high fidelity. The precise orientation of metal nanostructures can exhibit unique optical properties, via plasmonic phenomenon. As DNA origami enables the precise positioning of metal nanoparticles, it was successfully employed for chiral plasmonic nanostructures design [131]. Recently, 3D array of organic semiconductors was assembled using a DNA scaffold [132]. The aniline oligomers were incorporated into DNA structure and the reversible redox conversion between the pernigraniline and leucoemeraldine states was observed, suggesting presence of a viable electronic switch within the crystal.

In biochemical context the DNA nanotechnology is utilized for biosensing applications, such as molecular beacons [133,134], colorimetric detection systems based on AuNP-DNA conjugates [10,135], and three-dimensional nanocontainers [136]. The usage of DNA-nanoparticle conjugates has become practical in diagnostics, with a number of highly sensitive, colorimetric assays for detection of: proteins, metal ions and DNA, including the “bio-barcode” assay developed in Mirkin’s lab [137,138,139,140].

## 4. RNA—Gold Nanoparticles (AuNP) Conjugates

Ribonucleic acid differs from DNA in its tertiary folding and structural organization. Due to their natural regulatory function, RNA appears as an exceptional tool for targeted gene regulation, which is based on RNA interference (RNAi) mechanism [141]. Many different ways for RNA-dependent gene regulation have been proposed thus far, including the use of aptamers, siRNA and miRNA mimics [142]. Regulatory RNA structures can be adapted to aim against the gene of interest and synthesized to be finally applied to a host organism as a therapeutic agent.

**Figure 5:**
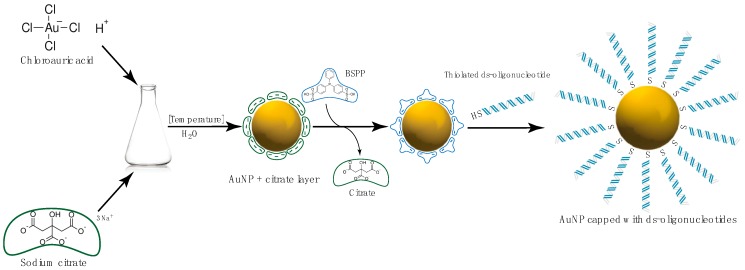
Schematic synthesis of thiol-linked spherical nucleic acids. Gold nanoparticles are synthesized from chloroauric acid in the presence of citrate; obtained AuNPs are treated with bis-(*p*-sulfonatophenyl)phenylphosphine (BSPP) to replace citrate ions prior to attachment of tiolated nucleic acids.

Metal nanoparticles, especially gold, have been successfully conjugated with RNA fragments and shown to cross the cellular membrane [143]. RNA can be conjugated with metal nanoparticle surface via thiol or electrostatic, non-covalent interactions. The stronger bond is however formed based on the thiol-metal interaction. In spherical nucleic acids (SNAs), DNA, or RNA component with the thiol group at its 5′-termini is introduced to the metal nanoparticle (for instance AuNP), where thiolated NA replace the citrate residues, originally used for stabilization of metallic nanoparticle (Figure 5). This type of interaction is prevalent in RNA-metal hybrids available thus far [143,144,145,146,147,148,149]. Other than thiol-based interactions are less common amongst the RNA-metal nanoparticles conjugates. These are usually electrostatic interactions between positive MeNP surfaces and RNA [150,151,152,153,154,155,156].

An interesting concept for RNAs hybridized with metal nanoparticles has been developed as the family of spherical ribonucleic acids (SRNA). SRNA, similarly to SNA, is composed of a metal nanoparticle core with ribonucleic acids fragments attached to the core surface. Such nanoparticles introduce the therapeutic potential of nucleic acids into the compact 3D nano-architecture, which allows for their application in variety of cells. First SNAs have been functionalized with DNA fragments, which provided with basic knowledge in the area of nucleic acids’ fusions [10,14]. However, due to the great regulatory capacity of RNA, the spherical ribonucleic acids (SRNA) have become a new way for gene expression regulation but were also recognized to act as biosensors, immunostimulatory and diagnostic agents. In SRNAs, RNA fragments are connected with AuNP via terminal thiol-metal interaction, that makes RNA accessible for enzymatic machinery and creates an opportunity for functional activity [143].

DNA and RNA fragments bind to endosomal toll-like receptors (TLRs), involved in innate immune system activation [157]. SNAs have been previously used as immunotherapeutics to stimulate immune response as well as hinder immunity in autoimmune disorders [158]. They have been exploited in immune cancer therapy [149], however, they are mostly used due to the cognate mRNA binding. Other materials may also serve as a platform for SNA formation, with the limitation being the choice of an appropriate linkage to form the hybrid [159]. Besides regulatory properties, the SRNAs can also be equipped with another functional components, like: fluorophores—used for detection or quantification; metal complexes—for imaging; chemical tags—for monitoring; antibody labels—for targeted delivery etc.

Most abundant use of SRNA utilizes the RNAi machinery to modulate gene expression, providing with a viable tool for treatment of unwanted protein-based malfunctions [144]. The RNAi relays on the machinery of proteins, including the endonuclease Dicer, which cleaves long double stranded RNA (dsRNA) to form siRNAs (18–22 bp). A siRNA is then introduced to the RNA-induced silencing complex (RISC), where it’s passenger strand is released and the guide strand serves as a recognition element for the target mRNA that is consequently cleaved by endonuclease [160]. Gene expression regulation with exogenous RNA regulatory fragments struggles with the major obstacles of tissue specificity and cellular transport. This is due to the hydrophobic nature of membranes and cellular defense mechanisms preventing external materials from entering the cell. To bypass this obstacle, researchers use transfecting agents, such as lipids, charged polymers, or vesicles. SRNA however, exhibit a potential to efficiently cross membranes without auxiliary transfection agents [143] and are reported to perform more effectively compared to classical delivery methods [161]. In the intracellular environment, they were shown not to stimulate immune response or cause toxicity while maintaining the regulatory potential of RNA. Simultaneously such setup can be applied to a wider range of cells and tissues, which could direct SRNA to become a tool in modern medicine.

It is important to bear in mind that the free siRNA in solution must be studied independently from the one grafted on the surface of metal nanoparticles [148].

The siRNA-AuNP conjugates may also be designed with an additional layer of cellular delivery agent [162]. Poly(β-amino ester)s are a common example to be used as a charge screening medium that undergo complexation with siRNA, and the complex can enter the cells. The RNA-AuNP hybrid was designed in such a way, that the gold nanoparticle was first capped with the PEG linker via thiol bond, then *N*-succinimidyl 3-(2-pyridyldithio)propionate linker was introduced, at which the disulphide bond with the HS-siRNA was formed.

Owing to their superior potential in cell permeability, SNAs have been also reported to cross blood-brain-barrier (BBB) that has always been a hurdle for onco-therapeutics [146,147]. In central nervous system (CNS), classical drug delivery methods are burdened with much lower stability and membrane permeability when applied intravenously or orally as they face the physical and physiological barriers of the tumor tissue [146,163]. The gold nanoparticles capped with regulatory RNAs were proven to maintain undegraded and successfully enter the tumor tissue leading to tumor reduction via RNAi machinery [146,147].

Another interesting application of RNA-AuNP conjugates is their topical delivery through skin. The epidermal growth factor receptor (EGFR)—targeted siRNA introduced into the SNA structure was investigated as a potential external treatment for skin diseases. Anti-EGFR SNA constructs, delivered externally, have successfully permeated mice’ integral skin and, after continued delivery, hindered expression of the EGFR and led reduction of epidermal thickness in treated cells [145]. That result reaffirms the efficacy of RNA–AuNP conjugates as a meek and easy approach for drug delivery, and their suitability to be delivered externally in ointment or other skin solution [145].

Another noteworthy potential application of RNA-AuNP conjugates appears in the field of biosensing and diagnostics. Among RNAs, aptamers, riboswitches or ribozymes are considered for biosensing due to their structural specificity, ligand recognition abilities and catalytic function.

Aptamers, in their binding specificity are similar to antibodies, feature a broad range of potential targets, like nucleic acids, proteins, or cells. The SELEX methodology has been used to create numerous target-specific aptamers [164]. Selected aptamer fragments may then be hybridized with AuNPs and modified to embody specific target. Gold nanoparticles, due to their surface plasmonic effect and high extinction coefficients, can be exploited as contrast agent. With the ability to conjugate aptamers with AuNP, it became possible to visualize individual elements of living cells using microscopic methods. For instance, the unique RNA aptamers were utilized as selective agents to target the prostate-specific membrane antigen (PSMA) [165]. The same aptamers were further applied in conjugation with AuNP and were shown to work well as target-specific imaging probes in the reflectance imaging [166]. In this case the thiolated DNA oligonucleotide linker was used to facilitate conjunction of AuNP and RNA aptamer. Similarly, the AuNP–RNA-aptamer conjugates were utilized in simultaneous treatment and CT imaging of the prostate cancer cells expressing PSMA [167]. The presented approach utilized: target specificity of the aptamer to deliver nanodevice to the prostate cancer cells, affinity of doxorubicin to GC-rich RNA duplexes so it could be introduced to the aptamer and optical properties of AuNP for imaging. The experiment showed that the proposed construct was able to deliver doxorubicin to the target cells, it could be visualized by CT and effectively killed cancer cells. Besides photo-detection methods were applied in conjunction with aptamers and gold nanoparticles, an electrochemical aptamer-based method for theophylline detection juxtaposes aptamers’ specificity with electrical properties of gold was used [168]. The RNA aptamer, in this case, was labeled with ferrocene and immobilized on the surface of gold electrode via thiol chemistry. In presented setup the ferrocene tag interacts with the surface of gold, when the aptamer is in the closed (ligand bound) position, hence boosting the electron transfer signal, which can be detected.

Riboswitches, discovered as RNA structural fragments naturally occurring in mRNA, have capability to response to protein, RNA, small molecules or ions binding [169]. When bound to the ligand, a riboswitch changes its spatial conformation or transforms. This prominent quality of RNA makes it an exceptional building block for biosensors. For example, a set of riboswitches, have been designed to specifically recognize between different molecules and applied to create an analytic RNA array. Riboswitches were immobilized via thiol chemistry on the surface of gold-coated plate and successfully used as a platform for detection of cobalt ions, secondary messengers, cGMP, cCMP, cAMP, flavin mononucleotide (FMN), and theophylline [170].

Similarly, a DNA–RNA–AuNP hybrid has been presented to serve as an RNA interference antagonistic biomimetic probe [171]. The probe allows to regulate the expression of the oncogene c-Myc via the RNAi and visualize the progress of the silencing process. Visualizing RISC’s activity in this process was possible thanks to the fluorescently labeled RNA fragment in the probe that is complementary to the innate Ago-miRNA complex.

A naturally occurring poly-A can preferentially attach on the surface of AuNPs, thus it may serve as an anchor for hybridizing nucleic acids. Such a design was already utilized in the AuNP–DNA hybrids [172,173] as well as with RNA. For example, a fluorescence-based method for Neomycin B detection, that bases on the poly-A linked RNA–AuNPs, has been successfully introduced [174]. The sensors were composed of AuNPs modified with self-assembling RNA aptamer, equipped with fluorescent probe. In this approach, binding the target molecule results in fluorescence quenching, which is a straight-forward and sensitive system for precise detection. An example of higher-order RNA structures/AuNP conjugates is phi29 phage pRNA that can be attached to the gold nanoparticles via thiol link and selectively connect to the protocapsid of the phage [175].

**Figure 6:**
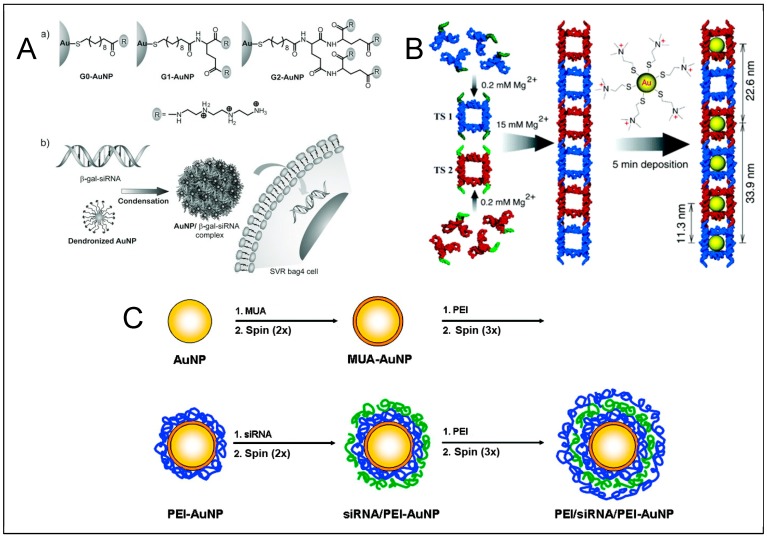
Examples of electrostatic RNA-gold nanoparticle interactions. (**A**) RNA entrapped within the dendronized polymer layer [152]. (**B**) Hierarchical supramolecular assembly of TS ladder decorated with cationic AuNPs [150]. (**C**) Layer by layer deposition of siRNA and poly(ethylene imine) (PEI) on the surface of AuNPs [151] (blue represents PEI, green represents siRNA). Taken with permission from: A [152], B [150], C [151].

Besides the thiol–gold bond the noncovalent interactions have also been exploited in such complex formation (Figure 6). Based on the electrostatic interaction, it became possible to coordinate RNA fragments on the surface of nanoparticle, without the need to modify the RNA structure (i.e., thiol). It was reported that such arrangements can be formed through layer by layer coating of AuNPs with oppositely charged electrolytes and were utilized for targeted gene silencing [151]. Additionally, nano-devices formed based off noncovalent forces embody different surface properties compared to typical SNA outline, which broadens applicability of RNA–metal hybrids. Such layered arrangements give a possibility to manipulate nanoparticles’ size and surface properties by experimental design, which, in return, gives raise to altered features of the nano-device. It is therefore another approach for controlled and non-toxic cell delivery of regulatory elements as well as a model for investigation.

An interesting system featuring electrostatic reciprocity of RNA and metallic nanoparticle rests on the fundament of AuNPs capped with dendronized ligands. Dendronized AuNPs with cumulative positive charge are able to attract and hold negatively charged RNA fragments, cross cell membrane and effectively silence the target gene without significant cytotoxicity [152]. Similar approach was utilized in delivery of siRNA targeted against c-Myc oncogene [153]. In this scenario branched polyethyleneimine was used as an attachment block to electrostatically connect siRNA. The construct was effectively delivered to cells and was proven to silence the gene expression.

The versatility of the AuNP nanoparticles allows for functionalization with many elements to make more complex and versatile constructs. A multifunctional construct, composed of AuNP capped with siRNA accompanied by adhesion and penetration promoting peptides was designed and reported to form an effective delivery system for both, in vitro and in vivo gene silencing [154]. In this study a combination of both semi-covalent and electrostatic binding was utilized to form a final device.

Another example of complex nanostructures based on AuNP was proposed as a potential tool for prostate cancer treatment via RNAi [155]. The presented nanodevices were equipped with either transferrin or folic acid that were shown to facilitate delivery of the construct to the cells (overexpressing receptors for those ligands) via receptor-mediated delivery. Those structures were further conjugated with siRNA fragments, as therapeutic elements, and applied to cells. The studied nanostructures were shown to reduce gene expression in the tested setup. It was therefore postulated that such device holds a promise to become a platform for targeted non-viral gene delivery vectors.

Slightly different approach may be undertaken to make AuNP-derived delivery devices, by displacing AuNP from the core. AuNPs can be trapped within the surface of dendrimers, to stabilize their 3D structure, which may be exploited as a delivery platform. Series of partially PEGylated poly(amidoamine) dendrimer-entrapped gold nanoparticles were proposed as another form of nucleic acid delivery means [156]. In such 3D configuration the presence of gold nanoparticles and PEG enhances gene delivery potential and lowers cytotoxic effect, compared to the dendrimer alone. The nanodevice was further conjugated with nucleic acid and effectively silenced the gene expression. Analogous approach was utilized in the glioblastoma cells study [176], where polyethylenimine-entrapped gold nanoparticles were used as cargo carriers. In that case, the dendrimer-gold complex concept was further improved by addition of the RGD peptide, to better the cell adhesion and was used as a delivery platform for siRNA for gene expression silencing. Such approaches encourage to be utilized as delivery vectors for various gene therapy applications.

Nature also provides with guidelines that can be followed in vitro. Naturally occurring RNA elements capable of forming higher-order structures have shown their potential to govern the formation of other metal particles. A set of naturally occurring RNA fragments have been synthesized, modified with piridyl groups and used for in vitro formation of palladium particles [177]. In that case, RNA fragments were speculated to participate in organometallic compounds recognition and stabilize the metallic intermediates in a sequence-dependent fashion.

Although the major significance of the RNA-metal complexes emerges in personalized medicine and RNAi therapy, these conjugates have found their place in other applications. Instead of identifying metal nanoparticles as core elements for RNA fragments’ to attach, the RNA may be treated as a platform for metal nanoparticle arrangement [150]. In particular, RNA’s structural flexibility, that has given rise to the RNA architectonics [178], may be further applied for precise arrangement of metal nanoparticles. In this case the electrostatic forces hold positively charged AuNPs within the tectoRNA geometry. Following this scheme, one can imagine, the possibility to create endless RNA–AuNP layouts in controlled fashion, which can potentially become a platform for modern nano-devices.

An alternative for double stranded siRNA based regulatory system is the tectoRNA [179]. RNA is well known for its ability to self-assemble into complex structural arrangements via tertiary interactions, that can be designed based off naturally occurring motifs [178]. Such RNA structures can be rationally designed and synthesized to act as a potential therapeutic agent. Over the years, since the development of RNA architectonics, many constructs have been generated and opened new routes for RNA-based biomedicine [180]. The most prevalently used methods for delivery of such therapeutic RNA are based on auxiliary agents, that are capable to cross the lipid bilayer. Such an approach, however, doesn’t assure the proper release of RNA, it’s stability or activity in target environment. It is therefore still essential to further explore drug delivery means that could replace the current imperfect methods. Clearly, in the field of RNA-based therapy, there is still plenty of room for improvement, especially in drug delivery, at which point, the variety of methods for conjugation of RNA and metal nanoparticles may offer another route to follow.

Taken all together, the RNA–metal interaction, emerges to be a valid fundament for modern nano-devices with a diversity of applications in science, biotechnology, personalized medicine and diagnostics.

## 5. Transport of DNA and RNA Conjugated Nanoparticles inside a Cell

It was demonstrated in numerous reports that metal-based nucleic acids-conjugated nanoparticles are able to enter inside the cells without additional carriers [75,181,182,183,184,185,186,187,188]. Nanoparticles from 6 to 200 nm in size access the tissue, where they initially accumulate in the extracellular matrix, and then penetrate the cells. The pathway that nanoparticles are transferred from extracellular matrix to specific subcellular compartments or organelles may be divided into several stages including:

1. Cell surface binding,
2. Translocation across the plasma membrane and thus penetration inside the cell (include membrane invagination and sorting into early endosomes)
3. Escape from endosomes or lysosomes
4. Subcellular localization [187,189].

Negatively charged cell membrane with the hydrophobic core is a barrier that requires appropriate features of absorbed molecules. The first stage of nanoparticle uptake is usually described as the electrostatic interactions between positively charged NP and the cell surface resembling electrostatic interactions exploited by cationic transfection agents [187].

Another way for the nanoparticles-cell surface interaction may be mediated by nonspecific adsorption of serum proteins onto the nanoparticle surface. Many of serum proteins are known to be absorbed by cells and may allow trafficking into the cells via cell surface receptors. It was shown, that the presence of serum proteins on the nanoparticles determines their intracellular half-life. Moreover, increasing the DNA density on the nanoparticle surface increases the number of adsorbed serum proteins [181,183,190,191]. Thus, serum proteins may be important for the internalization of gold nanoparticles [181], however some results clearly shown, that serum components may have the opposite effect and decrease cellular uptake of DNA–AuNPs [183,190]. The proposed mechanism assumes that in serum media, nanoparticles covering by serum proteins reduces the chance of nanoparticle interactions with scavenger receptors [183]. Furthermore, in certain cases, e.g., for receptor-targeting nanoparticles, the formation of a superficial protein layer on nanoparticle surface may disturb target recognition [191]. This can be prevented by nanoparticle surface functionalization with polyethylene glycol (PEG) [191]. Insertion of PEG molecules results in a reduction of non-specific serum protein–nanoparticle interactions and therefore restoration of target binding specificity [191].

It should be noted, that dominant factor in SNAs uptaken via membrane invaginations is a structure of surface oligonucleotides, not the metal core [185]. It was also demonstrated, that, not only properties of nanoparticles, but also cell type is crucial for that binding [182] and thus it was shown that non-cancerous cells are less sensitive to nanoparticle uptake, than cancer cells at in vitro conditions [187]. Furthermore, nanoparticle binding to the cell membrane may be enhanced by the surface functionalization using specific ligands, like peptides containing RGD motif, nucleolin, transferrin, EGF, or antibodies against membrane receptors [187].

The second stage of nanoparticle’s transfer—translocation across the plasma membrane—is an energy-dependent process and occurs mainly via endocytosis [185,187,192]. Internalization via endocytosis is also confirmed by the fact that nanoparticles inside the cell were found within vesicles [75,193]. Additionally, some data suggests, that gold nanoparticles may enter the cells via the scavenger receptor-mediated endocytosis pathway [181,183,185,194]. Scavenger receptors is a group of cell surface receptors known to mediate the endocytosis of polyanionic ligands, including nucleic acids [75,183].

The main mechanism of intracellular transport of DNA/RNA-conjugated nanoparticles is endocytosis. However, it was demonstrated, that endocytosis of different nanoparticles does not follow a single, specific route, but rather use multiple pathways including caveolae- or clathrin-mediated endocytosis as well as caveolae- and clathrin-independent endocytosis or macropinocytosis and even passive penetration or phagocytosis [182,185,192,195,196]. Moreover, it should be noted, that the mechanism of entry may be dependent on the nanoparticle’s specific properties, like size, surface, and shape and thus, while for DNA-coated gold nanoparticles named fPlas-gold the clathrin-mediated endocytosis seems to be dominant mechanism [75], for SNAs the involvement of this way is negligible [185]. Another internalization-limited factor is cell type, and thus caveolae mediated endocytosis, macropinocytosis and phagocytosis are excluded as the basic pathways of DNA-AuNPs entry into HeLa cells [183], while the main mechanism of SNAs uptake into C166 cells is the caveolae-mediated endocytosis [185].

**Figure 7:**
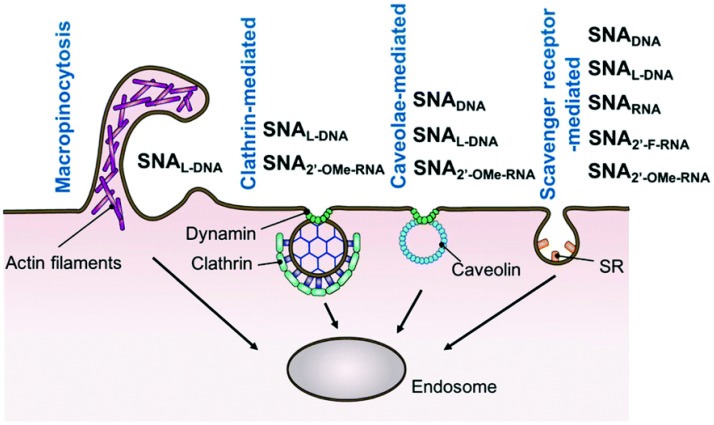
Schematic summary of the endocytosis mechanisms involved in the cellular uptake of spherical nucleic acids (SNAs). SR denotes the scavenger receptor. Taken with permission from [199].

Involvement of different pathways of cell entry may be caused by various factors such as type of nanoparticles and their physicochemical parameters (like size, surface charge, or composition), the topography of the oligonucleotide surface, presence of additional factors (e.g., serum proteins), as well as cell-type specific differences. For spherical nucleic acids, proposed mechanism assumes that upon binding to scavenger receptor, spherical nucleic acids are transported inside the cell via the caveolae-mediated pathway, presumably due to the close proximity of SNAs, SR-A, and lipid-raft microdomains [185]. For the most gold nanostructures, the receptor-mediated endocytosis (RME) is considered to be the major pathway for their cellular uptake [182,197]. During this process, upon the event of nanoparticle’s binding to the receptor, the cell membrane bends with a corresponding increase in elastic energy and decrease of configurational entropy. The immobilization of the bound receptors and diffusion to the wrapping site others, leads to complete membrane wrapping around the particle (Figure 7). This process is energy dependent and dramatically decreases in low temperature and at limited ATP [197]. On this account, it has been postulated that endocytosis is energetically unfavorable during delivery of small, charged nanoparticles inside cells. Thus, an alternative approach for AuNPs delivery should be employed, namely liposome-based delivery system, which provides an enhancement in the cellular uptake. AuNPs may be incorporated into the liposome particles or attached on their surfaces [198].

### 5.1. Intracellular Trafficking Organelle Distribution and Processing of Nanostructures

Inside the cell, nanoparticles are found mostly in the form of large clusters trapped in endosomes or lysosomes in the perinuclear region [75]. The maximum number of nanoparticles per cell depends on their sizes [181]. Data shown, that AuNP size, morphology, and surface modification are critical parameters not only for their cellular uptake, but also for delivery to organelles like nuclei or mitochondria [187]. Dynamics of cellular uptake and intracellular transport may vary depending on the nanoparticles type. It was shown, that SNAs could enter cells relatively fast, that after 5 min of incubation, each cell is associated with some NPs. After 15 min of incubation, individual SNAs are bound to cell membrane and invagination is started. The most abundant cell membrane binding occurs after 30 min incubation and cytosol localization of SNAs is visible within 2 h [185]. Other studies show that the fastest uptake of AuNPs is at initial 2 h of incubation, and after that time the rate of intracellular transport decreases and reaches plateau at 4–7 h, depending on nanoparticle size [181]. Among SNA containing five different oligonucleotides: DNA, L-DNA, RNA, 2′-methoxy-RNA (2′-OMe-RNA), and 2′-fluoro- RNA (2′-F-RNA), the cellular uptake efficiency was as follows: SNA2′-F-RNA > SNARNA > SNADNA > SNAL-DNA > SNA2′-OMe-RNA [199].

Co-localization studies shown, that nanoparticles from early endosomal vesicles through the late endosomes, were eventually transported to lysosomes [75]. In the case of DNA-coated gold nanoparticles fPlas-gold, the maximum level in early endosomes is observed after 2 h incubation, in late endosomes after 4 h and in lysosomes after 16 h [75].

After entering the cell, single nanoparticle trapped in endosomes moves along the microtubule. It is suggested that nanoparticles enter cells mainly in the form of single particles, however during intracellular trafficking individual nanoparticles are clustered. The intracellular transport of DNA-AuNPs may be divided into three phases based on their mobility [75]:

1. Low-motility particles (adhered to the membrane or bound to receptors)
2. High-motility particles (particles wrapped in early endosomal vesicles)
3. Low-motility particles (wrapped in late endosomes or lysosomes in the perinuclear region of the cell)

Moreover, it was demonstrated, that movement of fPlas-gold is critically dependent on their clustering states. The proposed three-stage transport for internalized fPlas-gold assumes that high-motility single particles are probably associated with microtubule-dependent transport mechanism, whereas movement of large clusters was diffusion-like [75]. Four types of intracellular transport of DNA-conjugated gold nanoparticles has been proposed [75]:

1. The fast-moving single particle chases a slow-moving one to merge (this type of motion is associated with vesicular fusion of early endosomes)
2. The single nanoparticle connects to small cluster on a different track, then rapidly separates and both molecules continue the moving along original tracks.
3. The small cluster moves rapidly towards another one, which is static and suddenly reverses its moving direction. This motion is similar to a dynein- and kinesin-based bidirectional cargo transport along the microtubule.
4. The two small clusters move along two tracks in the cross-section of the microtubules and one of them looses its mobility and becomes static while the second one departs with high speed.

The speed of encapsulated DNA–AuNPs transport is critically dependent on the size of clusters, however not on the types of organelle (endosomes and lysosomes) [75]. The mechanism cellular uptake and the role of nanoparticle clustering during endocytosis and intracellular traffic remain largely unexplored. After endocytosis, the main problem is to release nanoparticles from the endosomes and lysosomes. Nanoparticles are taken up, but trapped inside these organelles and thus are not active. To overcome this obstacle, some endosomal escape agents significantly supporting releasing of NPs into the cytoplasm might be used [200]. The nanoparticles trapped inside lysosomes exhibit slower diffusion time compared to endosomal NPs, which may be the result of the larger lysosome size [197]. AuNPs localization may be concentrated in subcellular compartments by specific surface functionalization. Targeting to specific organelles allows achieving the high local AuNP concentration and thus enables strengthening of their therapeutic activity and reducing side effects [187,189]. It should be noticed, that targeted and untargeted NPs seem to display different transport properties. Untargeted gold nanostructures display slower diffusion in the cell cytoplasm [197]. However multiple obstacles have to be overcome to achieve AuNPs accumulation in the desired cell compartment.

The process of elimination of nanoparticles from cells has not yet been thoroughly explained. Some reports shown, that nanoparticles are removed from the cells by exocytosis and this process occurs in a linear relationship to size [182,197], however the newest data suggest, that reduction of nanoparticles number inside cells is caused rather by mitosis, not exocytosis [201].

### 5.2. Physico-Chemical Properties of Nanoparticles and Their Cellular Uptake and Transport

Different sizes, shapes, and surface chemistries of metal nanoparticles significantly affect its cellular uptake and biological properties [181,193]. Both, shape (sphere, rod, cube, triangle, cage, etc.) and surface functionalities (i.e., type of DNA sequences exposed on the outer surface) of the non-targeted nano-assembly is responsible for differences in biological activity and nanoparticle’s processing [193,202]. It was demonstrated, that cellular uptake is heavily dependent upon the size of nanoparticles with the maximum penetration occurred for a nanoparticle size of 50 nm among the gold nanoparticles with sizes between 2 and 100 nm. Furthermore, not only the entering a cell, but also number of nanoparticles per vesicle is related to the size of the nanoparticle. Fact, that the gold nanoparticles (AuNPs) of diameter 40–50 nm demonstrate the highest cellular uptake may be caused by the shortest internalization time [181,197,203,204,205]. Furthermore, there are differences in the biodistribution of nanoparticles in the body depending on their size. Two hours after mice injection, AuNPs size 50 nm and more were shown to be localized mostly inside lung, liver and spleen, whereas smaller AuNPs were still present in the blood. Obtained results shown, that small nanoparticles have longer blood circulation half-lives compared to larger AuNPs [206,207].

The cellular uptake of nanoparticles is also dependent upon their shape. The uptake of rod-shaped AuNPs is slower than their spherical counterparts and among nanorods, longer particles enter cells slower than shorter AuNRs [181,197]. Moreover, it was shown, that nanoparticle size and shape can mediate receptor-ligand binding constants. Possible reason for these differences in uptake of the various AuNPs is, that the rod-shaped nanoparticles can have larger contact area with the cell membrane receptors than the spherical nanoparticles. Targeting ligands on the surface of the nanoparticle may be modified by changing its shape via specific nucleic acids. The spatial arrangement of oligonucleotides on the SNA surface cause, that these nanoparticles can bind strongly to the scavenger receptors, which mediate their cellular uptake [181,185,193,197]. Additionally, it was shown that one of the most important NP features for their transport inside the cells is their charge. The charge of nanoparticles is considered to be more important in the NP-uptake and intracellular transport than its size [197]. In case of chitosan-based nanoparticles, the cellular uptake rate and amount are both positively correlated with the surface charge, wherein some of positively charged NPs are able to escape from lysosome and exhibit perinuclear localization, whereas the negatively and neutrally charged NPs prefer to localize into lysosomes [208]. As it was elucidated, major effect on nanoparticle uptake has also interaction with serum proteins, which may mask the original surface and impact on the cellular uptake [197]. Summarizing, physico-chemical properties, including size, clustering, and surface chemistry of nanoparticles regulate their cellular uptake, transport and organelle distribution (Table 1).

## 6. Summary

Nanomaterials and nanoparticles express different functionalities, assembly patterns and behavior, compared to the macro world elements. Particularly interesting are complexes of metal nanoparticles with nucleic acids, which may be employed in different areas, such as medicine, diagnostics, or nano-electronics. The nucleic acids’ capacity to form 3D organized structures allows controllable assembly of two- and three-dimensional arrangements with metal nanoparticles to create crystalline forms and lattices. This holds a promise to deliver a building block for molecular machineries and nanomechanics, which can be programed to find and a specific cell in the body and shutdown a targeted process. The biocompatibility of gold nanoparticles and ease of functionalization with unlimited nucleic acids’ architectures, offers a wide range of potential applications. They emerge as a perfect fit for drug delivery and a perspective solution for personalized nanomedicine in the near future.

## Figures and Tables

**Table 1:** Comparison of gold nanoparticles toxicity and subcellular localization [202].

AuNP Size (nm)	Surface Group	Cell Line	Toxicity	Subcellular Localization	Reference
1, 4	Phospholipid	HeLa	Not reported	Lysosomal, perinuclear/nuclear	[198]
2, 8	Tat peptide	HTERT-BJ1	Low cytotoxicity below 10 µM	Cytoplasmic around the mitochondria and nuclear	[209]
3, 7	PEG	HeLa	Non toxic	Nuclear	[210]
5, 10, 15	CALNN, TAT and/or HA2 viral peptides	HeLa	Not reported	Cytoplasmic vesicles, lysosomal, endosomal, membranes	[211]
10	Oligonucleotides	HaCaT, A549, BALB/c 3T3, C166	Not reported	Cytoplasmic, endosomal	[185]
11–32	Nucleoplasmin	BALB/c 3T3 A31, MOP-8, SV-T2	Not reported	Nuclear and cytoplasmic	[212]
12	Sweet arrow peptide (SAP)	HeLa	Not reported	Endosomal	[213]
13	PEG	In vivo studies	Induction of acute inflammation and apoptosis	Cytoplasmic vesicles and lysosomal	[214]
16	PEG, CALNN, NLS, CPPs	HeLa	Not reported	Cytoplasmic, nuclear, lysosomal	[215]
20	citrate (Cit) compared with 11-mercaptoundecanoic acid (11-MUA)	HepG2	Non-toxic, DNA damage in Cit-AuNPS	Cytoplasmic, endosomal	[216]
20	BSA with NLS, receptor-mediated endocytosis peptides (RME)	HepG2	5% death	Nuclear	[189]
20	Biotinylated Tat-HA2, PEG-SH, anti-actin antibodies	BALB/c 3T3	Not reported	Cytoskeleton (cytoplasmic)	[217]
20–50	Citrate, PEG, CPP, Trastuzumab	DLD-1, SKOV-3, MDA-MB-231, SKBR-3, MCF-7	Cytotoxicity dependent on the surface group.	Intracellular	[218]
30–90	PEG	PC-3	Non toxic	Cytoplasmic and nuclear	[219]
14–100	Transferrin	STO, HeLa, SNB19, NPC	Non toxic	Endosomal	[181,182,220]
100	DPPE	C-32	Non toxic	Endosomal	[221]

## References

[1] Jeevanandam J., Barhoum A., Chan Y.S., Dufresne A., Danquah M.K. (2018). Review on nanoparticles and nanostructured materials: History, sources, toxicity and regulations. Beilstein J. Nanotechnol..

[2] Khan I., Saeed K., Khan I. (2019). Nanoparticles: Properties, applications and toxicities. Arab. J. Chem..

[3] Iravani S. (2011). Green synthesis of metal nanoparticles using plants. Green Chem..

[4] Khan Z.U., Khan A., Chen Y.M., Shah N.S., Muhammad N., Khan A.U., Tahir K., Khan F.U., Murtaza B., Ul Hassan S. (2017). Biomedical applications of green synthesized Nobel metal nanoparticles. J. Photochem. Photobiol. B.

[5] Meziani M.J., Sun Y.P. (2003). Protein-conjugated nanoparticles from rapid expansion of supercritical fluid solution into aqueous solution. J. Am. Chem. Soc..

[6] Piella J., Bastus N.G., Puntes V. (2017). Size-Dependent Protein-Nanoparticle Interactions in Citrate-Stabilized Gold Nanoparticles: The Emergence of the Protein Corona. Bioconjug. Chem..

[7] Draz M.S., Fang B.A., Zhang P.F., Hu Z., Gu S.D., Weng K.C., Gray J.W., Chen F.F. (2014). Nanoparticle-Mediated Systemic Delivery of siRNA for Treatment of Cancers and Viral Infections. Theranostics.

[8] Brigger I., Dubernet C., Couvreur P. (2002). Nanoparticles in cancer therapy and diagnosis. Adv. Drug Deliv. Rev..

[9] Azharuddin M., Zhu G.H., Das D., Ozgur E., Uzun L., Turner A.P.F., Patra H.K. (2019). A repertoire of biomedical applications of noble metal nanoparticles. Chem. Commun..

[10] Mirkin C.A., Letsinger R.L., Mucic R.C., Storhoff J.J. (1996). A DNA-based method for rationally assembling nanoparticles into macroscopic materials. Nature.

[11] Exicure. http://www.exicuretx.com/index.php.

[12] Cutler J.I., Auyeung E., Mirkin C.A. (2012). Spherical Nucleic Acids. J. Am. Chem. Soc..

[13] Li H., Zhang B., Lu X., Tan X., Jia F., Xiao Y., Cheng Z., Li Y., Silva D.O., Schrekker H.S. (2018). Molecular spherical nucleic acids. Proc. Natl. Acad. Sci. USA.

[14] Cutler J.I., Zhang K., Zheng D., Auyeung E., Prigodich A.E., Mirkin C.A. (2011). Polyvalent Nucleic Acid Nanostructures. J. Am. Chem. Soc..

[15] Connor E.E., Mwamuka J., Gole A., Murphy C.J., Wyatt M.D. (2005). Gold nanoparticles are taken up by human cells but do not cause acute cytotoxicity. Small.

[16] Yeh Y.C., Creran B., Rotello V.M. (2012). Gold nanoparticles: Preparation, properties, and applications in bionanotechnology. Nanoscale.

[17] Amendola V., MeneghettI M., Stener M., Guo Y., Chen S., Crespo P., Garcia M.A., Hernando A., Pengo P., Pasquato L. (2014). Physico-Chemical Characteristics of Gold Nanoparticles. Gold Nanoparticles in Analytical Chemistry.

[18] Amendola V., Pilot R., Frasconi M., Marago O.M., Iati M.A. (2017). Surface plasmon resonance in gold nanoparticles: A review. J. Phys. Condens. Matter.

[19] Das M., Shim K.H., An S.S.A., Yi D.K. (2011). Review on gold nanoparticles and their applications. Toxicol. Environ. Health Sci..

[20] Han G., You C.C., Kim B.J., Turingan R.S., Forbes N.S., Martin C.T., Rotello V.M. (2006). Light-regulated release of DNA and its delivery to nuclei by means of photolabile gold nanoparticles. Angew. Chem. Int. Ed..

[21] Weintraub K. (2013). The new gold standard. Nature.

[22] Hong R., Han G., Fernandez J.M., Kim B.J., Forbes N.S., Rotello V.M. (2006). Glutathione-mediated delivery and release using monolayer protected nanoparticle carriers. J. Am. Chem. Soc..

[23] Weissleder R. (2001). A clearer vision for in vivo imaging. Nat. Biotechnol..

[24] Sotnikov D.V., Berlina A.N., Ivanov V.S., Zherdev A.V., Dzantiev B.B. (2019). Adsorption of proteins on gold nanoparticles: One or more layers?. Colloids Surf. B Biointerfaces.

[25] Khashayar P., Amoabediny G., Larijani B., Hosseini M., Vanfleteren J. (2017). Fabrication and Verification of Conjugated AuNP-Antibody Nanoprobe for Sensitivity Improvement in Electrochemical Biosensors. Sci. Rep..

[26] Mustafaoglu N., Kiziltepe T., Bilgicer B. (2017). Site-specific conjugation of an antibody on a gold nanoparticle surface for one-step diagnosis of prostate specific antigen with dynamic light scattering. Nanoscale.

[27] Pissuwan D., Niidome T., Cortie M.B. (2011). The forthcoming applications of gold nanoparticles in drug and gene delivery systems. J. Control. Release.

[28] Camerin M., Magaraggia M., Soncin M., Jori G., Moreno M., Chambrier I., Cook M.J., Russell D.A. (2010). The in vivo efficacy of phthalocyanine-nanoparticle conjugates for the photodynamic therapy of amelanotic melanoma. Eur. J. Cancer.

[29] Labala S., Jose A., Venuganti V.V. (2016). Transcutaneous iontophoretic delivery of STAT3 siRNA using layer-by-layer chitosan coated gold nanoparticles to treat melanoma. Colloids Surf. B Biointerfaces.

[30] Mohammadi Z., Sazgarnia A., Rajabi O., Seilanian Toosi M. (2017). Comparative study of X-ray treatment and photodynamic therapy by using 5-aminolevulinic acid conjugated gold nanoparticles in a melanoma cell line. Artif. Cells Nanomed. Biotechnol..

[31] Huang X., Jain P.K., El-Sayed I.H., El-Sayed M.A. (2008). Plasmonic photothermal therapy (PPTT) using gold nanoparticles. Lasers Med. Sci..

[32] Kennedy L.C., Bickford L.R., Lewinski N.A., Coughlin A.J., Hu Y., Day E.S., West J.L., Drezek R.A. (2011). A new era for cancer treatment: Gold-nanoparticle-mediated thermal therapies. Small.

[33] Day E.S., Zhang L., Thompson P.A., Zawaski J.A., Kaffes C.C., Gaber M.W., Blaney S.M., West J.L. (2012). Vascular-targeted photothermal therapy of an orthotopic murine glioma model. Nanomedicine.

[34] Vines J.B., Yoon J.H., Ryu N.E., Lim D.J., Park H. (2019). Gold Nanoparticles for Photothermal Cancer Therapy. Front. Chem..

[35] D’Acunto M. (2018). Detection of Intracellular Gold Nanoparticles: An Overview. Materials.

[36] Huefner A., Septiadi D., Wilts B.D., Patel I.I., Kuan W.L., Fragniere A., Barker R.A., Mahajan S. (2014). Gold nanoparticles explore cells: Cellular uptake and their use as intracellular probes. Methods.

[37] Song K.H., Kim C., Maslov K., Wang L.V. (2009). Noninvasive in vivo spectroscopic nanorod-contrast photoacoustic mapping of sentinel lymph nodes. Eur. J. Radiol..

[38] Rogers N.J., Jeffery H.C., Claire S., Lewis D.J., Zikeli G., Hodges N.J., Egginton S., Nash G.B., Pikramenou Z. (2017). Tailoring iridium luminescence and gold nanoparticle size for imaging of microvascular blood flow. Nanomedicine.

[39] Bagheri S., Yasemi M., Safaie-Qamsari E., Rashidiani J., Abkar M., Hassani M., Mirhosseini S.A., Kooshki H. (2018). Using gold nanoparticles in diagnosis and treatment of melanoma cancer. Artif. Cells Nanomed. Biotechnol..

[40] Meola A., Rao J., Chaudhary N., Sharma M., Chang S.D. (2018). Gold Nanoparticles for Brain Tumor Imaging: A Systematic Review. Front. Neurol..

[41] Cruz L.J., Tacken P.J., Rueda F., Domingo J.C., Albericio F., Figdor C.G. (2012). Targeting nanoparticles to dendritic cells for immunotherapy. Methods Enzymol..

[42] Jia J., Zhang Y., Xin Y., Jiang C., Yan B., Zhai S. (2018). Interactions Between Nanoparticles and Dendritic Cells: From the Perspective of Cancer Immunotherapy. Front. Oncol..

[43] Surendran S.P., Moon M.J., Park R., Jeong Y.Y. (2018). Bioactive Nanoparticles for Cancer Immunotherapy. Int. J. Mol. Sci..

[44] Zhang D., Wu T., Qin X., Qiao Q., Shang L., Song Q., Yang C., Zhang Z. (2019). Intracellularly Generated Immunological Gold Nanoparticles for Combinatorial Photothermal Therapy and Immunotherapy against Tumor. Nano Lett..

[45] Ding Y., Jiang Z., Saha K., Kim C.S., Kim S.T., Landis R.F., Rotello V.M. (2014). Gold nanoparticles for nucleic acid delivery. Mol. Ther..

[46] Seeman N.C. (1982). Nucleic acid junctions and lattices. J. Theor. Biol..

[47] Fu T.J., Seeman N.C. (1993). DNA double-crossover molecules. Biochemistry.

[48] Sa-Ardyen P., Vologodskii A.V., Seeman N.C. (2003). The flexibility of DNA double crossover molecules. Biophys. J..

[49] Ding B.Q., Sha R.J., Seeman N.C. (2004). Pseudohexagonal 2D DNA crystals from double crossover cohesion. J. Am. Chem. Soc..

[50] Wang W., Lin T., Zhang S.Y., Bai T.X., Mi Y.L., Wei B.Y. (2016). Self-assembly of fully addressable DNA nanostructures from double crossover tiles. Nucleic Acids Res..

[51] LaBean T.H., Yan H., Kopatsch J., Liu F.R., Winfree E., Reif J.H., Seeman N.C. (2000). Construction, analysis, ligation, and self-assembly of DNA triple crossover complexes. J. Am. Chem. Soc..

[52] Liu D., Park S.H., Reif J.H., LaBean T.H. (2004). DNA nanotubes self-assembled from triple-crossover tiles as templates for conductive nanowires. Proc. Natl. Acad. Sci. USA.

[53] Wei B., Mi Y.L. (2005). A new triple crossover triangle (TXT) motif for DNA self-assembly. Biomacromolecules.

[54] Shen Z.Y., Yan H., Wang T., Seeman N.C. (2004). Paranemic crossover DNA: A generalized Holliday structure with applications in nanotechnology. J. Am. Chem. Soc..

[55] Liu W.Y., Wang X., Wang T., Sha R.J., Seeman N.C. (2008). PX DNA triangle oligomerized using a novel three-domain motif. Nano Lett..

[56] Winfree E., Liu F.R., Wenzler L.A., Seeman N.C. (1998). Design and self-assembly of two-dimensional DNA crystals. Nature.

[57] Yan H., Park S.H., Finkelstein G., Reif J.H., LaBean T.H. (2003). DNA-templated self-assembly of protein arrays and highly conductive nanowires. Science.

[58] Chen J., Seeman N.C. (1991). The Synthesis from DNA of a Molecule with the Connectivity of a Cube. Nature.

[59] Shih W.M., Quispe J.D., Joyce G.F. (2004). A 1.7-kilobase single-stranded DNA that folds into a nanoscale octahedron. Nature.

[60] Rothemund P.W. (2006). Folding DNA to create nanoscale shapes and patterns. Nature.

[61] Mao C.D., Sun W.Q., Shen Z.Y., Seeman N.C. (1999). A nanomechanical device based on the B-Z transition of DNA. Nature.

[62] Modi S., Swetha M.G., Goswami D., Gupta G.D., Mayor S., Krishnan Y. (2009). A DNA nanomachine that maps spatial and temporal pH changes inside living cells. Nat. Nanotechnol..

[63] Modi S., Nizak C., Surana S., Halder S., Krishnan Y. (2013). Two DNA nanomachines map pH changes along intersecting endocytic pathways inside the same cell. Nat. Nanotechnol..

[64] Zhou M.G., Liang X.G., Mochizuki T., Asanuma H. (2010). A Light-Driven DNA Nanomachine for the Efficient Photoswitching of RNA Digestion. Angew. Chem. Int. Ed..

[65] Lund K., Manzo A.J., Dabby N., Michelotti N., Johnson-Buck A., Nangreave J., Taylor S., Pei R.J., Stojanovic M.N., Walter N.G. (2010). Molecular robots guided by prescriptive landscapes. Nature.

[66] Chen Y., Wang M.S., Mao C.D. (2004). An autonomous DNA nanomotor powered by a DNA enzyme. Angew. Chem. Int. Ed..

[67] Yin P., Yan H., Daniell X.G., Turberfield A.J., Reif J.H. (2004). A unidirectional DNA walker that moves autonomously along a track. Angew. Chem. Int. Ed..

[68] Bath J., Green S.J., Turberfield A.J. (2005). A free-running DNA motor powered by a nicking enzyme. Angew. Chem. Int. Ed..

[69] Yurke B., Turberfield A.J., Mills A.P., Simmel F.C., Neumann J.L. (2000). A DNA-fuelled molecular machine made of DNA. Nature.

[70] Shin J.S., Pierce N.A. (2004). A synthetic DNA walker for molecular transport. J. Am. Chem. Soc..

[71] Tian Y., Mao C.D. (2004). Molecular gears: A pair of DNA circles continuously rolls against each other. J. Am. Chem. Soc..

[72] Douglas S.M., Bachelet I., Church G.M. (2012). A Logic-Gated Nanorobot for Targeted Transport of Molecular Payloads. Science.

[73] Andersen E.S., Dong M., Nielsen M.M., Jahn K., Subramani R., Mamdouh W., Golas M.M., Sander B., Stark H., Oliveira C.L.P. (2009). Self-assembly of a nanoscale DNA box with a controllable lid. Nature.

[74] Bujold K.E., Fakhoury J., Edwardson T.G.W., Carneiro K.M.M., Briard J.N., Godin A.G., Amrein L., Hamblin G.D., Panasci L.C., Wiseman P.W. (2014). Sequence-responsive unzipping DNA cubes with tunable cellular uptake profiles. Chem. Sci..

[75] Liu M., Li Q., Liang L., Li J., Wang K., Li J., Lv M., Chen N., Song H., Lee J. (2017). Real-time visualization of clustering and intracellular transport of gold nanoparticles by correlative imaging. Nat. Commun..

[76] Peng H.Y., Li X.F., Zhang H.Q., Le X.C. (2017). A microRNA-initiated DNAzyme motor operating in living cells. Nat. Commun..

[77] Rajendran A., Nakata E., Nakano S., Morii T. (2017). Nucleic-Acid-Templated Enzyme Cascades. ChemBioChem.

[78] Gelinas A.D., Davies D.R., Janjic N. (2016). Embracing proteins: Structural themes in aptamer-protein complexes. Curr. Opin. Struct. Biol..

[79] Sacca B., Niemeyer C.M. (2011). Functionalization of DNA nanostructures with proteins. Chem. Soc. Rev..

[80] Niemeyer C.M., Sano T., Smith C.L., Cantor C.R. (1994). Oligonucleotide-Directed Self-Assembly of Proteins—Semisynthetic DNA Streptavidin Hybrid Molecules as Connectors for the Generation of Macroscopic Arrays and the Construction of Supramolecular Bioconjugates. Nucleic Acids Res..

[81] Niemeyer C.M., Adler M., Gao S., Chi L.F. (2000). Supramolecular nanocircles consisting of streptavidin and DNA. Angew. Chem. Int. Ed..

[82] Li H.Y., Park S.H., Reif J.H., LaBean T.H., Yan H. (2004). DNA-templated self-assembly of protein and nanoparticle linear arrays. J. Am. Chem. Soc..

[83] Park S.H., Yin P., Liu Y., Reif J.H., LaBean T.H., Yan H. (2005). Programmable DNA self-assemblies for nanoscale organization of ligands and proteins. Nano Lett..

[84] Koyfman A.Y., Braun G.B., Reich N.O. (2009). Cell-Targeted Self-Assembled DNA Nanostructures. J. Am. Chem. Soc..

[85] Malo J., Mitchell J.C., Venien-Bryan C., Harris J.R., Wille H., Sherratt D.J., Turberfield A.J. (2005). Engineering a 2D protein-DNA crystal. Angew. Chem. Int. Ed..

[86] Aptagen. https://www.aptagen.com/apta-index/.

[87] Liu Y., Lin C.X., Li H.Y., Yan H. (2005). Protein nanoarrays—Aptamer-directed self-assembly of protein arrays on a DNA nanostructure. Angew. Chem. Int. Ed..

[88] Lin C.X., Katilius E., Liu Y., Zhang J.P., Yan H. (2006). Self-assembled signaling aptamer DNA arrays for protein detection. Angew. Chem. Int. Ed..

[89] Niemeyer C.M. (2001). Bioorganic applications of semisynthetic DNA-protein conjugates. Chem. A Eur. J..

[90] Niemeyer C.M. (2002). The developments of semisynthetic DNA-protein conjugates. Trends Biotechnol..

[91] Li H.Y., LaBean T.H., Leong K.W. (2011). Nucleic acid-based nanoengineering: Novel structures for biomedical applications. Interface Focus.

[92] Hu Q., Li H., Wang L., Gu H., Fan C. (2019). DNA Nanotechnology-Enabled Drug Delivery Systems. Chem. Rev..

[93] Yang D.Y., Campolongo M.J., Tran T.N.N., Ruiz R.C.H., Kahn J.S., Luo D. (2010). Novel DNA materials and their applications. Wires Nanomed. Nanobiotechnol..

[94] Seeman N., Sleiman H. (2017). DNA nanotechnology. Nat. Rev. Mater..

[95] Bath J., Turberfield A.J. (2007). DNA nanomachines. Nat. Nanotechnol.

[96] Liedl T., Sobey T.L., Simmel F.C. (2007). DNA-based nanodevices. Nano Today.

[97] Niemeyer C.M. (2007). Functional devices from DNA and proteins. Nano Today.

[98] Kwon Y.W., Lee C.H., Choi D.H., Jin J.I. (2009). Materials science of DNA. J. Mater. Chem..

[99] Carter J.D., LaBean T.H. (2011). Organization of Inorganic Nanomaterials via Programmable DNA Self-Assembly and Peptide Molecular Recognition. ACS Nano.

[100] Torimoto T., Yamashita M., Kuwabata S., Sakata T., Mori H., Yoneyama H. (1999). Fabrication of CdS nanoparticle chains along DNA double strands. J. Phys. Chem. B.

[101] Richter J., Seidel R., Kirsch R., Mertig M., Pompe W., Plaschke J., Schackert H.K. (2000). Nanoscale palladium metallization of DNA. Adv. Mater..

[102] Ford W.E., Harnack O., Yasuda A., Wessels J.M. (2001). Platinated DNA as precursors to templated chains of metal nanoparticles. Adv. Mater..

[103] Patolsky F., Weizmann Y., Lioubashevski O., Willner I. (2002). Au-nanoparticle nanowires based on DNA and polylysine templates. Angew. Chem. Int. Ed..

[104] Stoltenberg R.M., Woolley A.T. (2004). DNA-templated nanowire fabrication. Biomed. Microdevices.

[105] Alivisatos A.P., Johnsson K.P., Peng X.G., Wilson T.E., Loweth C.J., Bruchez M.P., Schultz P.G. (1996). Organization of ‘nanocrystal molecules’ using DNA. Nature.

[106] Le J.D., Pinto Y., Seeman N.C., Musier-Forsyth K., Taton T.A., Kiehl R.A. (2004). DNA-templated self-assembly of metallic nanocomponent arrays on a surface. Nano Lett..

[107] Pinto Y.Y., Le J.D., Seeman N.C., Musier-Forsyth K., Taton T.A., Kiehl R.A. (2005). Sequence-encoded self-assembly of multiple-nanocomponent arrays by 2D DNA scaffolding. Nano Lett..

[108] Sharma J., Chhabra R., Liu Y., Ke Y.G., Yan H. (2006). DNA-templated self-assembly of two-dimensional and periodical gold nanoparticle arrays. Angew. Chem. Int. Ed..

[109] Zhang J.P., Liu Y., Ke Y.G., Yan H. (2006). Periodic square-like gold nanoparticle arrays templated by self-assembled 2D DNA nanogrids on a surface. Nano Lett..

[110] Zheng J.W., Constantinou P.E., Micheel C., Alivisatos A.P., Kiehl R.A., Seeman N.C. (2006). Two-dimensional nanoparticle arrays show the organizational power of robust DNA motifs. Nano Lett..

[111] Nykypanchuk D., Maye M.M., van der Lelie D., Gang O. (2008). DNA-guided crystallization of colloidal nanoparticles. Nature.

[112] Park S.Y., Lytton-Jean A.K.R., Lee B., Weigand S., Schatz G.C., Mirkin C.A. (2008). DNA-programmable nanoparticle crystallization. Nature.

[113] Julin S., Nummelin S., Kostiainen M.A., Linko V. (2018). DNA nanostructure-directed assembly of metal nanoparticle superlattices. J. Nanoparticle Res..

[114] Mastroianni A.J., Claridge S.A., Alivisatos A.P. (2009). Pyramidal and Chiral Groupings of Gold Nanocrystals Assembled Using DNA Scaffolds. J. Am. Chem. Soc..

[115] Mitchell N., Schlapak R., Kastner M., Armitage D., Chrzanowski W., Riener J., Hinterdorfer P., Ebner A., Howorka S. (2009). A DNA Nanostructure for the Functional Assembly of Chemical Groups with Tunable Stoichiometry and Defined Nanoscale Geometry. Angew. Chem. Int. Ed..

[116] Sharma J., Chhabra R., Andersen C.S., Gothelf K.V., Yan H., Liu Y. (2008). Toward reliable gold nanoparticle patterning on self-assembled DNA nanoscaffold. J. Am. Chem. Soc..

[117] Ding B.Q., Deng Z.T., Yan H., Cabrini S., Zuckermann R.N., Bokor J. (2010). Gold Nanoparticle Self-Similar Chain Structure Organized by DNA Origami. J. Am. Chem. Soc..

[118] Pal S., Deng Z.T., Ding B.Q., Yan H., Liu Y. (2010). DNA-Origami-Directed Self-Assembly of Discrete Silver-Nanoparticle Architectures. Angew. Chem. Int. Ed..

[119] Helmi S., Ziegler C., Kauert D.J., Seidel R. (2014). Shape-Controlled Synthesis of Gold Nanostructures Using DNA Origami Molds. Nano Lett..

[120] Sun W., Boulais E., Hakobyan Y., Wang W.L., Guan A., Bathe M., Yin P. (2014). Casting inorganic structures with DNA molds. Science.

[121] Yan H., Zhang X.P., Shen Z.Y., Seeman N.C. (2002). A robust DNA mechanical device controlled by hybridization topology. Nature.

[122] Mao C.D., LaBean T.H., Reif J.H., Seeman N. (2000). Logical computation using algorithmic self-assembly of DNA triple-crossover molecules (vol 407, pg 493, 2000). Nature.

[123] Benenson Y., Paz-Elizur T., Adar R., Keinan E., Livneh Z., Shapiro E. (2001). Programmable and autonomous computing machine made of biomolecules. Nature.

[124] Fujibayashi K., Hariadi R., Park S.H., Winfree E., Murata S. (2008). Toward reliable algorithmic self-assembly of DNA tiles: A fixed-width cellular automaton pattern. Nano Lett..

[125] Wang B., Xie Y.J., Zhou S.H., Zhou C.J., Zheng X.D. (2017). Reversible Data Hiding Based on DNA Computing. Comput. Intell. Neurosci..

[126] Green S.J., Lubrich D., Turberfield A.J. (2006). DNA hairpins: Fuel for autonomous DNA devices. Biophys. J..

[127] Venkataraman S., Dirks R.M., Rothemund P.W.K., Winfree E., Pierce N.A. (2007). An autonomous polymerization motor powered by DNA hybridization. Nat. Nanotechnol..

[128] Lubrich D., Lin J., Yan J. (2008). A contractile DNA machine. Angew. Chem. Int. Ed..

[129] Deng Z.X., Mao C.D. (2004). Molecular lithography with DNA nanostructures. Angew. Chem. Int. Ed..

[130] Edwardson T.G.W., Lau K.L., Bousmail D., Serpell C.J., Sleiman H.F. (2016). Transfer of molecular recognition information from DNA nanostructures to gold nanoparticles. Nat. Chem..

[131] Kuzyk A., Schreiber R., Fan Z.Y., Pardatscher G., Roller E.M., Hogele A., Simmel F.C., Govorov A.O., Liedl T. (2012). DNA-based self-assembly of chiral plasmonic nanostructures with tailored optical response. Nature.

[132] Wang X., Sha R.J., Kristiansen M., Hernandez C., Hao Y.D., Mao C.D., Canary J.W., Seeman N.C. (2017). An Organic Semiconductor Organized into 3D DNA Arrays by “Bottom-up” Rational Design. Angew. Chem. Int. Ed..

[133] Tyagi S., Kramer F.R. (1996). Molecular beacons: Probes that fluoresce upon hybridization. Nat. Biotechnol..

[134] Drake T.J., Tan W.H. (2004). Molecular beacon DNA probes and their bioanalytical applications. Appl. Spectrosc..

[135] Mirkin C.A. (2000). Programming the assembly of two- and three-dimensional architectures with DNA and nanoscale inorganic building blocks. Inorg. Chem..

[136] Yang H., McLaughlin C.K., Aldaye F.A., Hamblin G.D., Rys A.Z., Rouiller I., Sleiman H.F. (2009). Metal-nucleic acid cages. Nat. Chem..

[137] Nam J.M., Thaxton C.S., Mirkin C.A. (2003). Nanoparticle-based bio-bar codes for the ultrasensitive detection of proteins. Science.

[138] Nam J.M., Stoeva S.I., Mirkin C.A. (2004). Bio-bar-code-based DNA detection with PCR-like sensitivity. J. Am. Chem. Soc..

[139] Stoeva S.I., Lee J.S., Thaxton C.S., Mirkin C.A. (2006). Multiplexed DNA detection with biobarcoded nanoparticle probes. Angew. Chem. Int. Ed..

[140] Zhang K.Y., Lv S.Z., Lin Z.Z., Li M.J., Tang D.P. (2018). Bio-bar-code-based photoelectrochemical immunoassay for sensitive detection of prostate-specific antigen using rolling circle amplification and enzymatic biocatalytic precipitation. Biosens. Bioelectron..

[141] Delihas N. (2015). Discovery and characterization of the first non-coding RNA that regulates gene expression, micF RNA: A historical perspective. World J. Biol. Chem..

[142] Setten R.L., Rossi J.J., Han S.P. (2019). The current state and future directions of RNAi-based therapeutics. Nat. Rev. Drug Discov..

[143] Giljohann D.A., Seferos D.S., Prigodich A.E., Patel P.C., Mirkin C.A. (2009). Gene regulation with polyvalent siRNA-nanoparticle conjugates. J. Am. Chem. Soc..

[144] Patel P.C., Hao L., Yeung W.S., Mirkin C.A. (2011). Duplex end breathing determines serum stability and intracellular potency of siRNA-Au NPs. Mol. Pharm..

[145] Zheng D., Giljohann D.A., Chen D.L., Massich M.D., Wang X.Q., Iordanov H., Mirkin C.A., Paller A.S. (2012). Topical delivery of siRNA-based spherical nucleic acid nanoparticle conjugates for gene regulation. Proc. Natl. Acad. Sci. USA.

[146] Jensen S.A., Day E.S., Ko C.H., Hurley L.A., Luciano J.P., Kouri F.M., Merkel T.J., Luthi A.J., Patel P.C., Cutler J.I. (2013). Spherical nucleic acid nanoparticle conjugates as an RNAi-based therapy for glioblastoma. Sci. Transl. Med..

[147] Kim H.J., Takemoto H., Yi Y., Zheng M., Maeda Y., Chaya H., Hayashi K., Mi P., Pittella F., Christie R.J. (2014). Precise engineering of siRNA delivery vehicles to tumors using polyion complexes and gold nanoparticles. ACS Nano.

[148] Barnaby S.N., Lee A., Mirkin C.A. (2014). Probing the inherent stability of siRNA immobilized on nanoparticle constructs. Proc. Natl. Acad. Sci. USA.

[149] Kranz L.M., Diken M., Haas H., Kreiter S., Loquai C., Reuter K.C., Meng M., Fritz D., Vascotto F., Hefesha H. (2016). Systemic RNA delivery to dendritic cells exploits antiviral defence for cancer immunotherapy. Nature.

[150] Koyfman A.Y., Braun G., Magonov S., Chworos A., Reich N.O., Jaeger L. (2005). Controlled spacing of cationic gold nanoparticles by nanocrown RNA. J. Am. Chem. Soc..

[151] Elbakry A., Zaky A., Liebl R., Rachel R., Goepferich A., Breunig M. (2009). Layer-by-layer assembled gold nanoparticles for siRNA delivery. Nano Lett..

[152] Kim S.T., Chompoosor A., Yeh Y.C., Agasti S.S., Solfiell D.J., Rotello V.M. (2012). Dendronized gold nanoparticles for siRNA delivery. Small.

[153] Shaat H., Mostafa A., Moustafa M., Gamal-Eldeen A., Emam A., El-Hussieny E., Elhefnawi M. (2016). Modified gold nanoparticles for intracellular delivery of anti-liver cancer siRNA. Int. J. Pharm..

[154] Conde J., Rosa J., de la Fuente J.M., Baptist P.V. (2013). Gold-nanobeacons for simultaneous gene specific silencing and intracellular tracking of the silencing events. Biomaterials.

[155] Guo J.F., O’Driscoll C.M., Holmes J.D., Rahme K. (2016). Bioconjugated gold nanoparticles enhance cellular uptake: A proof of concept study for siRNA delivery in prostate cancer cells. Int. J. Pharm..

[156] Hou W.X., Wei P., Kong L.D., Guo R., Wang S.G., Shi X.Y. (2016). Partially PEGylated dendrimer-entrapped gold nanoparticles: A promising nanoplatform for highly efficient DNA and siRNA delivery. J. Mater. Chem. B.

[157] Majer O., Liu B., Barton G.M. (2017). Nucleic acid-sensing TLRs: Trafficking and regulation. Curr. Opin. Immunol..

[158] Radovic-Moreno A.F., Chernyak N., Mader C.C., Nallagatla S., Kang R.S., Hao L.L., Walker D.A., Halo T.L., Merkel T.J., Rische C.H. (2015). Immunomodulatory spherical nucleic acids. Proc. Natl. Acad. Sci. USA.

[159] Barnaby S.N., Perelman G.A., Kohlstedt K.L., Chinen A.B., Schatz G.C., Mirkin C.A. (2016). Design Considerations for RNA Spherical Nucleic Acids (SNAs). Bioconjug. Chem..

[160] Agrawal N., Dasaradhi P.V.N., Mohmmed A., Malhotra P., Bhatnagar R.K., Mukherjee S.K. (2003). RNA interference: Biology, mechanism, and applications. Microbiol. Mol. Biol. R.

[161] Hao L., Patel P.C., Alhasan A.H., Giljohann D.A., Mirkin C.A. (2011). Nucleic acid-gold nanoparticle conjugates as mimics of microRNA. Small.

[162] Lee J.S., Green J.J., Love K.T., Sunshine J., Langer R., Anderson D.G. (2009). Gold, poly(beta-amino ester) nanoparticles for small interfering RNA delivery. Nano Lett..

[163] Allhenn D., Boushehri M.A., Lamprecht A. (2012). Drug delivery strategies for the treatment of malignant gliomas. Int. J. Pharm..

[164] Zhuo Z., Yu Y., Wang M., Li J., Zhang Z., Liu J., Wu X., Lu A., Zhang G., Zhang B. (2017). Recent Advances in SELEX Technology and Aptamer Applications in Biomedicine. Int. J. Mol. Sci..

[165] Lupold S.E., Hicke B.J., Lin Y., Coffey D.S. (2002). Identification and characterization of nuclease-stabilized RNA molecules that bind human prostate cancer cells via the prostate-specific membrane antigen. Cancer Res..

[166] Javier D.J., Nitin N., Levy M., Ellington A., Richards-Kortum R. (2008). Aptamer-targeted gold nanoparticles as molecular-specific contrast agents for reflectance imaging. Bioconjug. Chem..

[167] Kim D., Jeong Y.Y., Jon S. (2010). A Drug-Loaded Aptamer-Gold Nanoparticle Bioconjugate for Combined CT Imaging and Therapy of Prostate Cancer. ACS Nano.

[168] Ferapontova E.E., Olsen E.M., Gothelf K.V. (2008). An RNA aptamer-based electrochemical biosensor for detection of theophylline in serum. J. Am. Chem. Soc..

[169] Garst A.D., Edwards A.L., Batey R.T. (2011). Riboswitches: Structures and Mechanisms. CSH Perspect. Biol..

[170] Seetharaman S., Zivarts M., Sudarsan N., Breaker R.R. (2001). Immobilized RNA switches for the analysis of complex chemical and biological mixtures. Nat. Biotechnol..

[171] Zhang K., Yang X.J., Zhao W., Xu M.C., Xu J.J., Chen H.Y. (2017). Regulation and imaging of gene expression via an RNA interference antagonistic biomimetic probe. Chem. Sci..

[172] Jiang H.Y., Ling K., Tao X.J., Zhang Q.Q. (2015). Theophylline detection in serum using a self-assembling RNA aptamer-based gold nanoparticle sensor. Biosens. Bioelectron..

[173] Yao G.B., Pei H., Li J., Zhao Y., Zhu D., Zhang Y.N., Lin Y.F., Huang Q., Fan C.H. (2015). Clicking DNA to gold nanoparticles: Poly-adenine-mediated formation of monovalent DNA-gold nanoparticle conjugates with nearly quantitative yield. NPG Asia Mater..

[174] Ling K., Jiang H., Zhang L., Li Y., Yang L., Qiu C., Li F.R. (2016). A self-assembling RNA aptamer-based nanoparticle sensor for fluorometric detection of Neomycin B in milk. Anal. Bioanal. Chem..

[175] Moll W.D., Guo P. (2007). Grouping of ferritin and gold nanoparticles conjugated to pRNA of the phage phi29 DNA-Packaging motor. J. Nanosci. Nanotechnol..

[176] Kong L.D., Qiu J.R., Shi X.Y. (2017). Multifunctional PEI-entrapped gold nanoparticles enable efficient delivery of therapeutic siRNA into glioblastoma cells. J. Control. Release.

[177] Gugliotti L.A., Feldheim D.L., Eaton B.E. (2005). RNA-mediated control of metal nanoparticle shape. J. Am. Chem. Soc..

[178] Jaeger L., Westhof E., Leontis N.B. (2001). TectoRNA: Modular assembly units for the construction of RNA nano-objects. Nucleic Acids Res..

[179] Grabow W.W., Zakrevsky P., Afonin K.A., Chworos A., Shapiro B.A., Jaeger L. (2011). Self-assembling RNA nanorings based on RNAI/II inverse kissing complexes. Nano Lett..

[180] Jedrzejczyk D., Gendaszewska-Darmach E., Pawlowska R., Chworos A. (2017). Designing synthetic RNA for delivery by nanoparticles. J. Phys.Condens. Matter.

[181] Chithrani B.D., Ghazani A.A., Chan W.C.W. (2006). Determining the size and shape dependence of gold nanoparticle uptake into mammalian cells. Nano Lett..

[182] Chithrani B.D., Chan W.C.W. (2007). Elucidating the mechanism of cellular uptake and removal of protein-coated gold nanoparticles of different sizes and shapes. Nano Lett..

[183] Patel P.C., Giljohann D.A., Daniel W.L., Zheng D., Prigodich A.E., Mirkin C.A. (2010). Scavenger Receptors Mediate Cellular Uptake of Polyvalent Oligonucleotide-Functionalized Gold Nanoparticles. Bioconjug. Chem..

[184] Wang Z.D., Zhang J.Q., Ekman J.M., Kenis P.J.A., Lu Y. (2010). DNA-Mediated Control of Metal Nanoparticle Shape: One-Pot Synthesis and Cellular Uptake of Highly Stable and Functional Gold Nanoflowers. Nano Lett..

[185] Choi C.H.J., Hao L.L., Narayan S.P., Auyeung E., Mirkin C.A. (2013). Mechanism for the endocytosis of spherical nucleic acid nanoparticle conjugates. Proc. Natl. Acad. Sci. USA.

[186] Liang H., Zhang X.B., Lv Y., Gong L., Wang R., Zhu X., Yang R., Tan W. (2014). Functional DNA-containing nanomaterials: Cellular applications in biosensing, imaging, and targeted therapy. Acc. Chem. Res..

[187] Kodiha M., Wang Y.M., Hutter E., Maysinger D., Stochaj U. (2015). Off to the Organelles—Killing Cancer Cells with Targeted Gold Nanoparticles. Theranostics.

[188] Nemati H., Ghahramani M.H., Faridi-Majidi R., Izadi B., Bahrami G., Madani S.H., Tavoosidana G. (2017). Using siRNA-based spherical nucleic acid nanoparticle conjugates for gene regulation in psoriasis. J. Control. Release.

[189] Tkachenko A.G., Xie H., Coleman D., Glomm W., Ryan J., Anderson M.F., Franzen S., Feldheim D.L. (2003). Multifunctional gold nanoparticle-peptide complexes for nuclear targeting. J. Am. Chem. Soc..

[190] Giljohann D.A., Seferos D.S., Patel P.C., Millstone J.E., Rosi N.L., Mirkin C.A. (2007). Oligonucleotide loading determines cellular uptake of DNA-modified gold nanoparticles. Nano Lett..

[191] Dai Q., Walkey C., Chan W.C.W. (2014). Polyethylene Glycol Backfilling Mitigates the Negative Impact of the Protein Corona on Nanoparticle Cell Targeting. Angew. Chem. Int. Ed..

[192] Panzarini E., Mariano S., Carata E., Mura F., Rossi M., Dini L. (2018). Intracellular Transport of Silver and Gold Nanoparticles and Biological Responses: An Update. Int. J. Mol. Sci..

[193] Ohta S., Glancy D., Chan W.C.W. (2016). DNA-controlled dynamic colloidal nanoparticle systems for mediating cellular interaction. Science.

[194] Mokhtarzadeh A., Vahidnezhad H., Youssefian L., Mosafer J., Baradaran B., Uitto J. (2019). Applications of Spherical Nucleic Acid Nanoparticles as Delivery Systems. Trends Mol. Med..

[195] Li H.Y., Chen Z., Ho L.W., Chan P.S., Li Q., Leung S.C., Zhang B., Lai K.L., Kwon G.S., Choi C.H.J. (2017). Oligonucleotide-conjugated nanoparticles for targeted drug delivery via scavenger receptors class A: An in vitro assessment for proof-of-concept. Int. J. Pharm..

[196] Wang H., Chen B.B., He M., Li X.T., Chen P.Y., Hu B. (2019). Study on uptake of gold nanoparticles by single cells using droplet microfluidic chip-inductively coupled plasma mass spectrometry. Talanta.

[197] Chithrani D.B. (2010). Intracellular uptake, transport, and processing of gold nanostructures. Mol. Membr. Biol..

[198] Chithrani D.B., Dunne M., Stewart J., Allen C., Jaffray D.A. (2010). Cellular uptake and transport of gold nanoparticles incorporated in a liposomal carrier. Nanomed. Nanotechnol..

[199] Song W.C., Kim K.R., Park M., Lee K.E., Ahn D.R. (2017). Backbone-modified oligonucleotides for tuning the cellular uptake behaviour of spherical nucleic acids. Biomater. Sci..

[200] Dowdy S.F. (2017). Overcoming cellular barriers for RNA therapeutics. Nat. Biotechnol.

[201] Bourquin J., Septiadi D., Vanhecke D., Balog S., Steinmetz L., Spuch-Calvar M., Taladriz-Blanco P., Petri-Fink A., Rothen-Rutishauser B. (2019). Reduction of Nanoparticle Load in Cells by Mitosis but Not Exocytosis. ACS Nano.

[202] Levy R., Shaheen U., Cesbron Y., See V. (2010). Gold nanoparticles delivery in mammalian live cells: A critical review. Nano Rev..

[203] Aoyama Y., Kanamori T., Nakai T., Sasaki T., Horiuchi S., Sando S., Niidome T. (2003). Artificial viruses and their application to gene delivery. size-controlled gene coating with glycocluster nanoparticles. J. Am. Chem. Soc..

[204] Jiang W., Kim B.Y.S., Rutka J.T., Chan W.C.W. (2008). Nanoparticle-mediated cellular response is size-dependent. Nat. Nanotechnol..

[205] Osaki F., Kanamori T., Sando S., Sera T., Aoyama Y. (2004). A quantum dot conjugated sugar ball and its cellular uptake. On the size effects of endocytosis in the subviral region. J. Am. Chem. Soc..

[206] Cho W.S., Cho M., Jeong J., Choi M., Han B.S., Shin H.S., Hong J., Chung B.H., Jeong J., Cho M.H. (2010). Size-dependent tissue kinetics of PEG-coated gold nanoparticles. Toxicol. Appl. Pharm..

[207] Dong Y.C., Hajfathalian M., Maidment P.S.N., Hsu J.C., Naha P.C., Si-Mohamed S., Breuilly M., Kim J., Chhour P., Douek P. (2019). Effect of Gold Nanoparticle Size on Their Properties as Contrast Agents for Computed Tomography. Sci. Rep..

[208] Yue Z.G., Wei W., Lv P.P., Yue H., Wang L.Y., Su Z.G., Ma G.H. (2011). Surface Charge Affects Cellular Uptake and Intracellular Trafficking of Chitosan-Based Nanoparticles. Biomacromolecules.

[209] de la Fuente J.M., Berry C.C. (2005). Tat peptide as an efficient molecule to translocate gold nanoparticles into the cell nucleus. Bioconjug. Chem.

[210] Gu Y.J., Cheng J.P., Lin C.C., Lam Y.W., Cheng S.H., Wong W.T. (2009). Nuclear penetration of surface functionalized gold nanoparticles. Toxicol. Appl. Pharm..

[211] Cesbron Y., Shaheen U., Free P., Levy R. (2015). TAT and HA2 facilitate cellular uptake of gold nanoparticles but do not lead to cytosolic localisation. PLoS ONE.

[212] Feldherr C.M., Lanford R.E., Akin D. (1992). Signal-mediated nuclear transport in simian virus 40-transformed cells is regulated by large tumor antigen. Proc. Natl. Acad. Sci. USA.

[213] Pujals S., Bastus N.G., Pereiro E., Lopez-Iglesias C., Punte V.F., Kogan M.J., Giralt E. (2009). Shuttling Gold Nanoparticles into Tumoral Cells with an Amphipathic Proline-Rich Peptide. Chembiochem.

[214] Cho W.S., Cho M.J., Jeong J., Choi M., Cho H.Y., Han B.S., Kim S.H., Kim H.O., Lim Y.T., Chung B.H. (2009). Acute toxicity and pharmacokinetics of 13 nm-sized PEG-coated gold nanoparticles. Toxicol. Appl. Pharm..

[215] Nativo P., Prior I.A., Brust M. (2008). Uptake and intracellular fate of surface-modified gold nanoparticles. ACS Nano.

[216] Fraga S., Faria H., Soares M.E., Duarte J.A., Soares L., Pereira E., Costa-Pereira C., Teixeira J.P., de Lourdes Bastos M., Carmo H. (2013). Influence of the surface coating on the cytotoxicity, genotoxicity and uptake of gold nanoparticles in human HepG2 cells. J. Appl. Toxicol..

[217] Kumar S., Harrison N., Richards-Kortum R., Sokolov K. (2007). Plasmonic nanosensors for imaging intracellular biomarkers in live cells. Nano Lett..

[218] Cruz E., Kayser V. (2019). Synthesis and Enhanced Cellular Uptake In Vitro of Anti-HER2 Multifunctional Gold Nanoparticles. Cancers.

[219] Malugin A., Ghandehari H. (2010). Cellular uptake and toxicity of gold nanoparticles in prostate cancer cells: A comparative study of rods and spheres. J. Appl. Toxicol..

[220] Yang P.H., Sun X.S., Chiu J.F., Sun H.Z., He Q.Y. (2005). Transferrin-mediated gold nanoparticle cellular uptake. Bioconjug. Chem..

[221] Soman N., Marsh J., Lanza G., Wickline S. (2008). New mechanisms for non-porative ultrasound stimulation of cargo delivery to cell cytosol with targeted perfluorocarbon nanoparticles. Nanotechnology.

